# WALKING THE TALK: ENGAGING PATIENTS AND CAREGIVERS AS COLLABORATORS IN PALLIATIVE CARE RESEARCH

**DOI:** 10.1093/geroni/igz038.1015

**Published:** 2019-11-08

**Authors:** Anna N Rahman, Susan Enguidanos

**Affiliations:** 1 University of Southern California, Leonard Davis School of Gerontology, Los Angeles, California, United States; 2 University of Southern California, Los Angeles, California, United States

## Abstract

Palliative care is inherently patient directed and family oriented. Given this, it is especially important that patients and caregivers participate in developing new services intended to meet their needs. Our experience with conducting a controlled trial of home-based palliative care demonstrates this point. This poster will describe the implementation and dissemination decisions that patient and caregiver stakeholders guided during a comparative effectiveness trial of palliative care. It also will present strategies for engaging stakeholders and identify the impact of these strategies on continuous study improvement. For the study, we convened an advisory committee (AC), which included eight patients and caregivers among its 34 members. These eight partners in particular helped us identify meaningful patient- and caregiver-reported outcome measures, Throughout the study’s 2.5-year period, they offered suggestions that influenced the tone and topics addressed in quarterly AC meetings. They also helped refine the study’s patient and caregiver assessment instruments by roleplaying with our research assistants. When we launched a Google group to promote discussion among AC members, patient and caregiver stakeholders were especially forthcoming. They identified gaps in care and consumer understanding of palliative care that we otherwise would have overlooked. Of note, our stakeholders provided input on how to best to introduce a new palliative care program to patients. These engagement strategies create feedback loops that open new opportunities for continuously improving research. Moreover, engagement of patients and caregivers in research planning and implementation provides important and otherwise overlooked perspectives while ensuring a patient-centered focus, consistent with palliative care goals.

